# Judgment and Observation

**Published:** 1910-04-30

**Authors:** 


A Journal of

The Medical Sciences and Hospital Administration.

New Series. No. 167, Vol. VII. [No. 1239, Vol. XLVIII.] SATURDAY, APRIL 30, 1910.

### Judgment and Observation.

Many have been the attempts to define genius and reduce it to some tangible formula by which the more ordinary mortals might judge it. Talent, on the other hand, is more susceptible of analysis, and seems to consist of an equipoise between the powers of observation and judgment. Genius requires at least a third ingredient—imagination. To confine ourselves, however, to men of talent who have become, or who are likely to become, famous in their own particular department. Although talented men are met with quite often, it is surprising to notice how few, comparatively speaking, of those whose powers of observation are excellent, can be relied on to draw sound conclusions from their own observations. To give but one example of inaccurate judgment, we may cite the case of the Cephalodiscus brought back by the *Challenger* Expedition. This was correctly observed to possess two dark spots which were judged at the time to be eyes. Subsequent investigation, however, proved that these were merely the apertures of the oviducts. The field naturalist is *par excellence* a man with acutely trained powers of observation. His knowledge of various animals, insects, and plants as they exist in nature is, in some cases, astonishing, but he seldom passes from the particular to the general, and if he does he is generally in error. But if the field naturalist lacks the power of seizing new associations and of co-ordinating his vast and special knowledge, the scientist who spends most of his days in the laboratory generally suffers from an excessive development of the " eye of faith." A detailed account, for example, of the reproduction of *Monocystis* was supplied by Wolters and accepted generally, so that it actually found its way into text-books. But no other investigator has yet been able to confirm these detailed observations. Even in the preparation of so excellent a work as the account of the experiments conducted in the Wellcome Research Laboratory of Khartoum, provided as it was with excellent illustrations, there were inaccuracies in the representation of the number of chromosomes in the plates devoted to trypanosomes. It seems, indeed, as if, owing to the increasing bustle of life, intelligent anticipation is to become a national code of life, and the consequences will be by no means satisfactory.

It would, perhaps, be useful for those engaged in microscopic investigations to invite the aid of some perfect tyro who might gaze upon their preparations and tell them exactly what was visible to the untrained eye. As a corrective it would not be without its uses. It seems probable that our present system of scientific education is largely at fault, while at the same time questionable whether the uniformity of teaching is not pushed too far. Given an aptitude for observation, it can be trained; given a power of right judgment, it can be ameliorated. But the question remains: can either of these faculties be produced by education? Nature study is all very well. Books are being produced by the dozen in which nice little descriptions are given of pond life or stream life, with plentiful illustrations, and various papers endeavour to praise these serious attempts to instruct the youthful mind. But too much is written nowadays. The pleasure of all scientific work, be it in the field or in the laboratory, lies in the pursuit alone, not in reading about it, and it is better that at first the youthful investigator should fall into error rather than find everything cut and dried for him, with all the pitfalls carefully wired round and all the traps accurately screened.

It is not the quantity of scientific investigations that we want, but the quality. Every little man who takes a course of chemistry at a night school becomes, in his own imagination, a scientist. Every field naturalist who can call a daisy *Bellis perennis* is satisfied that he too belongs to a great and respected body which attempts to dispel the ignorance of the masses. The point on which we would insist is not that these peculiarities are in themselves at all reprehensible, but that they do not deserve the talk and publicity which they have obtained. If Darwin, who is on all sides accounted a perfect example of real scientific genius, waited twenty years before publishing his " Origin of Species," we do not think that a couple of centuries or so of oblivion would do much harm to the modern scientologist.

